# Supplementary material for: Matrix Metalloproteinase-1 (MMP1) Upregulation through Promoter Hypomethylation Enhances Tamoxifen Resistance in Breast Cancer
Source: Cancers (Basel). 2022 Feb 27;14(5):1232. doi: 10.3390/cancers14051232 (PMC8909089; doi:10.3390/cancers14051232)

Matrix Metalloproteinase-1 (MMP1) Upregulation through Promoter Hypomethylation Enhances Tamoxifen Resistance in Breast Cancer

Hyeon Woo Kim, Jae Eun Park, Minjae Baek, Heejoo Kim, Hwee Won Ji, Sung Hwan Yun, Dawoon Jeong, Juyeon Ham, Sungbin Park, Xinpei Lu, Han-Sung Kang, and Sun Jung Kim

Supporting Information

Table S1. Primer sequences used for qRT-PCR and MSP employed in this study

Gene	Forward primer (5´→ 3´)	Reverse primer (5´→ 3´)	Supplier
**qRT-PCR**			
GAPDH	ACATCGCTCAGACACCATG	TGTAGTTGAGGTCAATGAAGGG	IDT
MMP1	GACAGATTCTACATGCGCAC	CCCTTTGAAAAACCGGACTTC	Bioneer
**MSP**			
MMP1 M	ATTTTAAATAAGATGTGTGCG	AACCATCAAAACCAATCTTTT	Bioneer
MMP1 U	ATTTTAAATAAGATGTGTGTG	AACCATCAAAACCAATCTTTT	

Figure S1. Uncropped Western blots. The boxed areas in the images are shown in the main text.

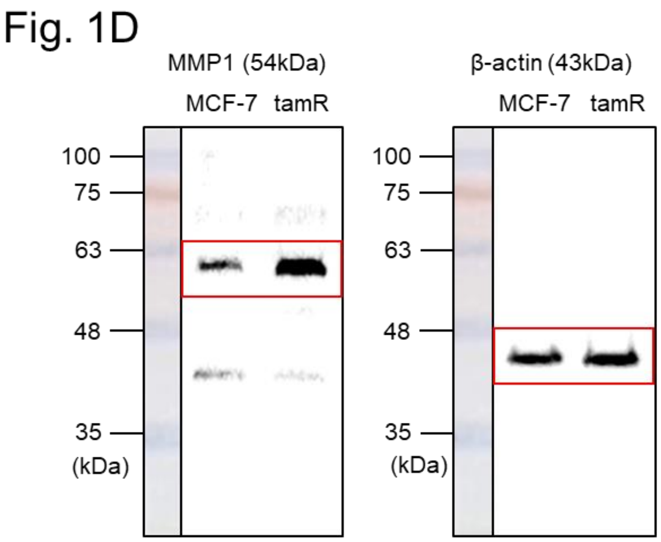

cell line	MCF-7	MCF-7/tamR
β-actin	18466.85	22235.32
MMP1	9789.24	26143.85

Figure S2. Induction of downregulation of MMP1 in the MCF-7 and MCF-7/tamR cells. Stable MMP1 knockdown cells [MCF-7/tamR/shMMP1 (A) and MCF-7/shMMP1 (B)] were developed using two shRNAs that target different MMP1 sites (#1 and #2). Expression of MMP1 was then evaluated by qRT-PCR. shNC; control shRNA. All results are reported as the means ± SE of three independent experiments. *$P < 0.01$

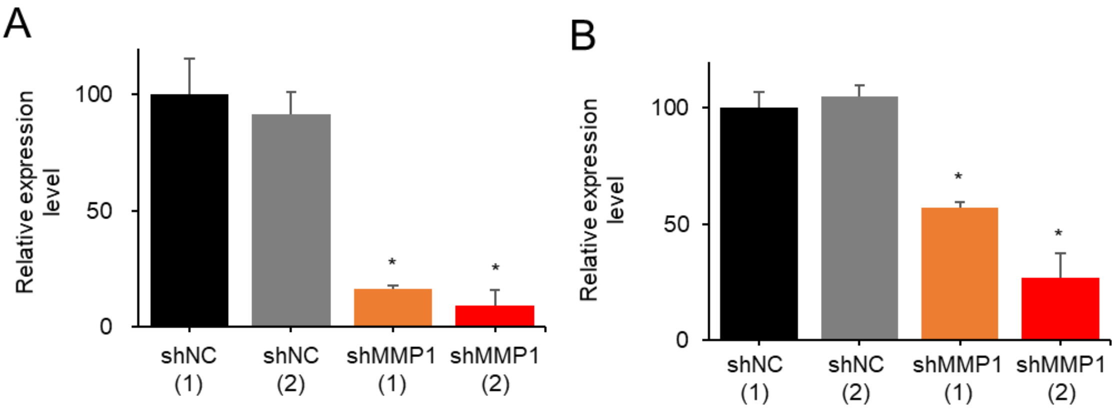

Supplement: Supplementary file 1 [file cancers-14-01232-s001.zip › cancers-1585863-supplementary.pdf]
